# Family systemic therapy: intervention in autism spectrum disorder

**DOI:** 10.1192/j.eurpsy.2024.1479

**Published:** 2024-08-27

**Authors:** C. Díaz Mayoral, P. Setién Preciados, E. Arroyo Sánchez, J. Gimillo Bonaque

**Affiliations:** Psiquiatría, Hospital Universitario Príncipe de Asturias, Madrid, Spain

## Abstract

**Introduction:**

Autism spectrum disorder (ASD) is a neurodevelopmental disorder characterized by communication impairments and difficulties in social interaction. These impairments can affect relationships with family members, increase stress and frustration for both the patient and family members, and contribute to behavioral disturbances in these patients. They are frequently associated with high rates of psychiatric comorbidity.

**Objectives:**

Given the impact of this disorder on the family unit, we set out to assess the clinical effectiveness of systemic family therapy, its influence on improving communication and coping with this disorder, strengthening relationships and mental health in these patients and their families.

**Methods:**

A literature review was performed by searching for articles in Pubmed on May 24, 2023, focusing the terminology used on “Autism Spectrum Disorder” and “Systemic Family Therapy”. The search was limited to full text articles in English and Spanish, published in the last 10 years.

**Results:**

Several authors have stated that systemic family therapy could be beneficial:

- Providing education.
- Reporting additional educational resources.
- Focusing sessions on improving social and communication skills, mood and coping behaviors.
- Providing therapy to all family members to cope with this disorder and what it implies. It will be very important that the patient with ASD understands his condition and can receive support from his family, working with siblings on the bonding and coping with this condition.
- Contributing to facilitate mourning the loss of the condition of “neurotypical” person, exploring emotions, feelings and belief systems, valuing the social and cultural context of the family.

**Conclusions:**

Reviewing a variety of literature on this therapeutic approach, the authors concluded that “strategic, narrative and structural interventions can be applied from multiple approaches, especially suited to the challenges often faced by patients with ASD and their families”. Therapeutic work needs to involve different family members at different times. Therapeutic conversations will consider the child, the family and the family unit in context. Studies so far have not been able to establish whether particular systemic approaches have more favorable outcomes than others, which warrants further research.

**Disclosure of Interest:**

None Declared

